# Establishing injury surveillance in emergency departments in Nepal: protocol for mixed methods prospective study

**DOI:** 10.1186/s12913-020-05280-9

**Published:** 2020-05-18

**Authors:** Dan Magnus, Santosh Bhatta, Julie Mytton, Elisha Joshi, Emma L. Bird, Sumiksha Bhatta, Sunil Raja Manandhar, Sunil Kumar Joshi

**Affiliations:** 1 Centre for Academic Child Health, University of Bristol, Bristol, UK; 2 Centre for Academic Child Health, University of the West of England, Bristol, UK; 3 Nepal Injury Research Centre, Kathmandu Medical College Public Limited, Kathmandu, Nepal; 4 Mother and Infant Research Activities, Kathmandu, Nepal

**Keywords:** Trauma, Public health, Urgent care, Hospital, LMIC

## Abstract

**Background:**

Globally, injuries cause more than 5 million deaths annually, a similar number to those from HIV, Tuberculosis and Malaria combined. In people aged between 5 and 44 years of age trauma is the leading cause of death and disability and the burden is highest in low- and middle-income countries (LMICs). Like other LMICs, injuries represent a significant burden in Nepal and data suggest that the number is increasing with high morbidity and mortality. In the last 20 years there have been significant improvements in injury outcomes in high income countries as a result of organised systems for collecting injury data and using this surveillance to inform developments in policy and practice. Meanwhile, in most LMICs, including Nepal, systems for routinely collecting injury data are limited and the establishment of injury surveillance systems and trauma registries have been proposed as ways to improve data quality and availability.

**Methods:**

This study will implement an injury surveillance system for use in emergency departments in Nepal to collect data on patients presenting with injuries. The surveillance system will be introduced in two hospitals and data collection will take place 24 h a day over a 12-month period using trained data collectors. Prospective data collection will enable the description of the epidemiology of hospital injury presentations and associated risk factors. Qualitative interviews with stakeholders will inform understanding of the perceived benefits of the data and the barriers and facilitators to embedding a sustainable hospital-based injury surveillance system into routine practice.

**Discussion:**

The effective use of injury surveillance data in Nepal could support the reduction in morbidity and mortality from adult and childhood injury through improved prevention, care and policy development, as well as providing evidence to inform health resource allocation. This study seeks to test a model of injury surveillance based in emergency departments and explore factors that have the potential to influence extension to additional settings.

## Background

Globally, injuries cause more than 5 million deaths annually – a similar number to those from HIV/AIDS, Tuberculosis and Malaria combined [1]. Injuries are the leading cause of death in people aged 5–24 years globally [1, 2] and the leading cause of disability for people aged between 5 and 44 years resulting in long term physical, psychological and financial difficulty [3]. The burden is highest in low and middle income countries (LMICs), which account for approximately 90% of global injury-related deaths [4, 5].

Almost one third of injuries leading to death globally occur as a result of violence, with other main causes including road traffic collisions, falls and drowning [1]. Road traffic injuries are a particular problem worldwide and are recognised as the eighth leading cause of disability adjusted life years, projected to rise to fifth by 2030 [2, 6]. Nearly 1.3 million people die each year on the world’s roads and it is worth noting that LMICs account for just 54% of the world’s vehicles but more than 90% of global road traffic deaths [6].

Similar to other LMICs, injuries represent a significant burden in Nepal [7]. An estimated 13,500 to 18,000 injury-related deaths occur in Nepal each year, with a further 780,000 to 1,000,000 Disability Adjusted Life Years (DALYs) attributed to injuries [8]. Road traffic collisions are a common cause of trauma in Nepal [9, 10] and police data suggest that the number is increasing annually with high morbidity and mortality [11, 12]. Household survey data suggest that other injuries, occurring in and around the home, such as falls and burns, poisoning, occupational and animal related injuries also contribute heavily to Nepal’s injury burden [13, 14].

In the last 20 years there have been significant improvements in trauma outcomes in high income countries as a result of organised systems for collecting trauma data and using this surveillance to inform developments in policy and practice [15]. Despite this, the quality of data relating to childhood and adult injury is inversely correlated to where the greatest problems exist. The existing data on trauma in most LMICs remains poor with injury data coming from low income countries constituting around 1% of all data [16]. The use of injury surveillance systems and trauma registries has been proposed as a way to improve this. Injury surveillance is “the ongoing and systematic collection, analysis, interpretation and dissemination of health information” [17]. The collection and use of data on risk factors, incidence, severity, outcomes, and costs can assist practitioners and researchers in identifying populations at risk, implementing and evaluating prevention programs, and formulating and evaluating policy [17]. By comparison trauma registries provide timely, accurate, and comprehensive data to inform the delivery of care to injured patients [18]. This study has been designed to inform injury prevention activities and policy development and is therefore focussed on surveillance.

There are a number of examples of LMICs establishing trauma registries in hospitals for surveillance purposes, for example in Kenya [19], Nigeria [20] and Uganda [21]. However the data from LMIC trauma registries is more at risk than in high income countries of being incomplete and inaccurately coded due to a lack of data collection infrastructure and support [16]. Compared to evidence emerging from Africa, there is a relative lack of surveillance data from the Indian subcontinent, although data from a few small surveillance studies carried out in India [22] and Fiji [23, 24] have indicated the feasibility of collecting injury data in real time.

There are no formal injury surveillance systems running in Nepal and the current level of routine data collection on injuries in Nepali emergency departments is limited to that routinely collected through the Hospital Management Information System (HMIS). Through the HMIS data are collated manually at the local level and uploaded electronically to a central system each month. Data are predominantly counts to establish the frequency of different conditions treated. Along with minimal demographic data there is minimal routine data collected on mechanism of injury to inform decisions on vulnerable groups or opportunities for injury prevention.

The existing literature provides limited evidence on recommendations for establishing injury data collection systems in low resource settings, though a number of recent systematic reviews have looked specifically at the implementation of trauma registries [25–27]. One review, identified four elements that should be considered for trauma registry implementation [26]: the identification of a local champion (and possibly paid data collectors); the establishment of a process of accountability; an electronic system for data collection and analysis of relevant data/outcomes; and a data quality auditing mechanism to ensure the validity of the data collected. To support the development of evidence-based injury prevention and pre-hospital care in Nepal, the establishment of functioning hospital-based injury surveillance systems are an important first step.

This programme of work aims to develop and assess injury surveillance data collection tools and processes to serve as the foundations for an injury surveillance system in Nepal.

## Methods/design

### Aim

To develop, introduce and assess a hospital-based injury surveillance tool to explore its potential for wider use in Nepal.

## Objectives

(i) Designing of an injury surveillance tool and data collection process
(ii) Prospective collection of data on all injuries presenting to two hospital emergency departments over 12 months
(iii) Process evaluation to explore the barriers and facilitators to establishing an ongoing injury surveillance system

### Research design

This is a prospective mixed methods study incorporating the collection of quantitative data to describe the epidemiology of injuries presenting to the study sites over a period of 12 months and qualitative data to inform a process evaluation conducted during the last 3 months of injury surveillance.

### Study setting

The Makwanpur district of Nepal was selected as the study site because of its geographical location, composition of its population and its varied terrains. It contains high hills, mid hills and plains which are characteristic of many districts in Nepal. These geographical features are important because they relate to injury risk and the results of the injury surveillance work in this area may make the results applicable to many areas in the rest of Nepal. Makwanpur district has an estimated population of 420,477 living in 86,127 households, with an average number of household occupants of 4.88, and 83% of the population living in rural areas. The district has an area of 2426 km^2^, making up 1.6% of the total land area of Nepal. It contains 78 different ethnic groups with their own languages and cultures. The major ethnicities are Tamang (indigenous), Brahmin, Chhetri, Magar, and marginalised groups such as Praja/Chepang, and endangered groups such as Bankariya [28].

Both study hospitals are secondary care hospitals in Hetauda, a sub-metropolitan city of Makwanpur district and the temporary province headquarter, approximately 120 km south-east of Kathmandu. Hetauda is located at the country’s major highway intersection, between the east-west (Mahendra) and north-south (Tribhuvan) highways [28]. Most local injury cases attend these hospitals because of the long distances to other major tertiary care hospitals and a lack of adequate transportation systems. Both hospitals have the facilities to provide treatment for major and minor trauma [29]. Hetauda hospital is a government-funded district hospital with 110 beds serving about 300 emergency and outpatient attendances per day. There are 19 doctors and 47 clinical staff comprising paramedics and nurses. Chure Hill Hospital is a private hospital with 25 beds serving about 60 emergency and outpatient attendances per day. There are 12 doctors and 65 clinical staff comprising paramedics and nurses (source: verbal inquiry with the hospital management authority).

### Inclusion and exclusion

All types of injury are within the scope of the prospective surveillance component of this study if meeting the inclusion and exclusion criteria (Table 1), including intentional and unintentional injuries. The terms intentional and unintentional denote whether an injury was meant to harm the victim or not. Intentional injuries include suicide and self-harm, homicide, assault and child abuse or purposeful neglect. Unintentional injuries are those without any intent of self-harm, homicide, or suicide and include, for example; falls, road traffic collisions, poisoning, burns and scalds, and animal related injuries (bite, sting, crush or attack) [30]. For this study an injury death is defined as any death resulting from an injury defined above and occurring within 7 days of the injury.

**Table 1:** Criteria for sample selection

Inclusion criteria	Exclusion criteria
1. Adults or children presenting for urgent care at a participating site, with ***a new history of*** injury of any severity including: a. Multisystem injury b. Isolated injuries c. Toxic ingestions/poisonings d. Subacute injuries e. Burns or scald g. Drowning or near drowning h. Choking, strangulation or suffocation i. Patients deceased on arrival due to injury 2. Adult or children referred from another regional clinic or site for further management of injuries or medical condition	1. Repeated attendance in the same (emergency) department for the same injury 2. Previous attendance in other study site hospital for the same injury 3. Injury sustained > 7 days prior to presentation

### Study setting and sample size

The study setting is people who have sustained a new injury of any kind presenting to either of the two study hospitals within 7 days of the injury event. Data will be collected from patients presenting with injuries between 1st of April 2019 and 31st March 2020 inclusive. No sample size estimation is required as this is a prospective observational study of all cases of children and adults presenting with injuries who meet the inclusion criteria. Information from the Government of Nepal Department of Health Services for Hetauda Hospital in the year 2016/2017 revealed a total of approximately 21,000 emergency department patient visits. Similar data were not available for the emergency department at Chure Hill Hospital. From these data we estimated there may be 50 patients per day presenting to each of the hospital sites equating to 100 patients per day in total, with approximately one third of these attendances for injuries. The study team will expect to see around 30 injuries per day across the two sites and about 10,000 injury cases over the course of 12 months.

### Data collection

#### Surveillance data

A standardised data collection form has been developed drawing on a number of existing tools including the African Federation of Emergency Medicine [31], the World Health Organisation [17] and the Home and Leisure Accident and Surveillance System (HASS) from the Royal Society for the Prevention of Accidents [32], developed and adapted for the Nepal context. The data collection form will not replace existing clinical records as the existing clinical record keeping in the two study sites are not sufficiently detailed or reliable to capture the required epidemiological or clinical data on injuries. Through the process evaluation we will capture views on the potential to integrate a surveillance system into clinical records, such as recommended by the WHO Global Alliance for the Care of the Injured [6]. Once urgent clinical care has been given and the patient is stable, data collectors will approach the patient (or carer, where necessary and appropriate) for consent to record information. Anonymised patient data will be collected on socio-demographics, date of injury, mechanism of injury, clinical presentation, diagnosis and disposition (discharge, admission, referral or death). Data will be captured electronically onto tablet computers preloaded with the data collection tool using REDCap (Research Electronic Data Capture) software [33, 34]. Each case will be given a unique filename. Data will be encrypted and uploaded to the secure online REDCap database when internet connection is available.

**Fig. 1:**
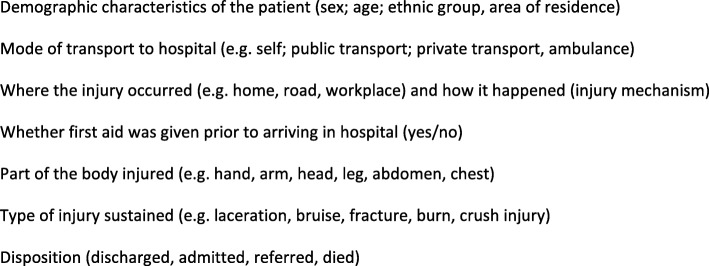
Data variables that will be collected

**Fig. 2:**
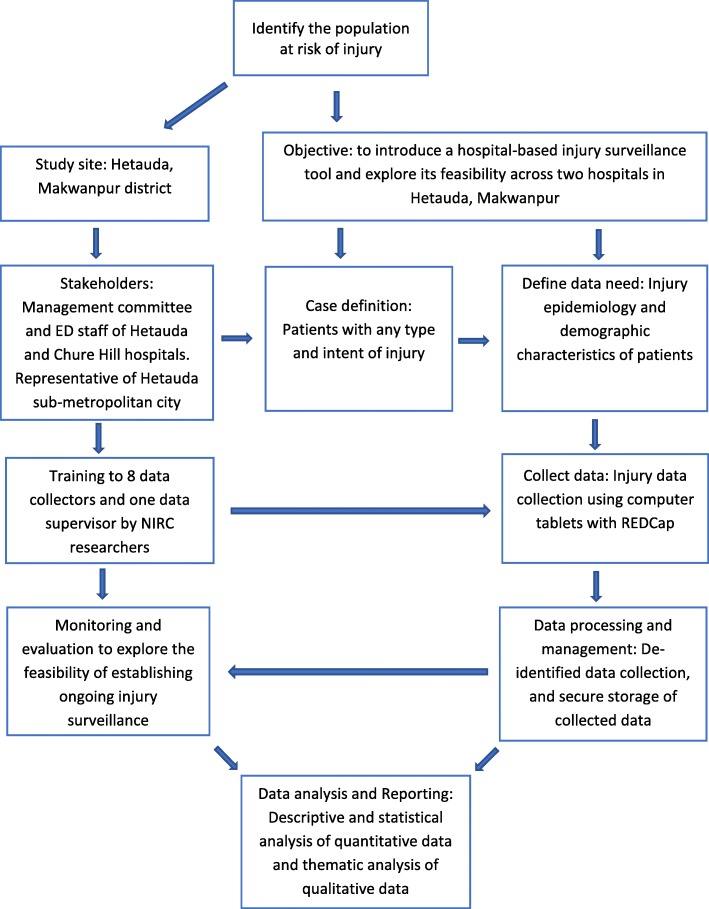
Conceptual framework for this hospital-based injury surveillance study (modified injury surveillance framework adapted from WHO 2001)

Data collectors for this study have a health-related background and are recruited, employed and paid by Mother and Infant Research Activities (MIRA), a non-governmental organisation working in health research in Nepal since 1994 (http://www.mira.org.np/mira/). A total of 11 staff (five data collectors for Hetauda Hospital, 4 data collectors for Chure Hill Hospital, one supervisor and one data quality officer) have been recruited and trained to use the data collection tools and to complete the quality assurance processes. As data collection at the two hospital sites will be for 24 h a day, 7 days a week, a full-shift rota has been designed to enable sufficient coverage for data collection whilst also allowing sufficient rest and optimum working conditions for staff. Data variables to be collected are summarised in Fig. 1 and the conceptual framework for this study is presented in Fig. 2.

#### Pretesting and training

The data collection tool was pre-tested in Hetauda and Chure Hill Hospitals and any data issues rectified before formal data collection commenced. This took the following format for the two study sites:

(i) Training for data collectors on using the data collection tool

All data collectors and the study supervisor were provided with a full day training workshop that included: the background to the project; the inclusion/exclusion criteria for identifying an eligible case; the technical use of the REDCap surveillance tool; detail on what information should be collected and how it should be recorded; ethical and governance issues relating to the surveillance tool. Written definitions for each study variable and guidance on how to complete the data collection form were also produced in a data dictionary to support the training of data collection staff, and for use as a reference document during the data collection process when required. The data collectors recruited had good spoken and written English and training was provided largely in Nepali with some English and provision of the data dictionary, training materials and the REDCap data tool in both English and Nepali translations.

(ii) Supported practice using the surveillance tool

Following training, data collectors were supervised to collect and upload data using the injury surveillance tool in the clinical environment. This stage of training provided experience of using the tool and helped data collectors to familiarise themselves with working in the clinical environment. Technical support was on hand from the project team to answer any questions raised by the data collectors.

(iii) Feedback from the practice sessions and tool modification

Following the training, data collectors had the opportunity to feedback comments to the project team and the team collated problems or issues arising during the practice phase, and minor modifications to the tool and data dictionary were made.

#### Quality assurance

To ensure adequate data quality, the following mechanisms will be used:

1. **Study log –** Each case entered onto REDCap will be listed on a study log. One (paper) log sheet will be completed for each 24-h period in each site. It will be completed by the data collector on shift and will be compared with the ED register once a day to ensure ‘missing’ patients are recorded. The log will be submitted to the data supervisor each day.
2. **Case ascertainment** – The data supervisor will examine the Emergency Department register at each study hospital daily and compare this with the study log. This process will occur retrospectively every 24 h during the week and following the weekend/holidays. Any patients missing from the study log will be recorded anonymously on REDCap, and given a unique ID number, with a summary of any details pertaining to the case that can be identified from the ED register. Monitoring will take place weekly and a monthly report of the number of missing cases will be sent to the study team.
3. **Data validation –** This will be assessed by undertaking sampled, double data entry in each hospital every 8 weeks. This will involve two data collectors each completing a surveillance form electronically for the same patient with subsequent comparison of the completed forms by the data supervisor. Levels of agreement will be recorded on a ‘data quality’ audit form. The reason for using an additional form in this way is to ensure that the quality of the data entry is monitored and reported by the study team and used to inform ongoing training and supervision of the data collectors.

#### Qualitative data collection

We will interview emergency department staff, senior hospital managers and data collectors either face-to-face or by telephone, to identify facilitators and barriers to the implementation of the tool at the two study sites and seek their views regarding the potential to expand the surveillance programme to additional sites across Nepal. Interviewees will be provided with a participant information sheet and have the opportunity to ask questions prior to deciding whether or not to participate in an interview. Participants will be asked to sign a consent form where interviews are conducted face to face, or a verbal consent will be recorded digitally for telephone interviews. A semi-structured interview will be completed using a topic guide to explore: program goals/organization, how implementation of the surveillance system transpired, enablers and barriers to complete and accurate data capture, as well as perceived impact on the larger trauma system and future recommendations for injury surveillance. Interviews will be conducted by trained researchers, and recorded using a digital, recording device where consent given.

### Data management and analysis

#### Surveillance data

Anonymised data will be entered into REDCap and the dataset will be backed up daily. Access to the data is via an encrypted SSL website providing different levels of access for different staff (for example data collectors can enter data, while researchers can enter, edit and extract data). REDCap maintains an audit trail of all actions including data changes made to the system. The final non-identifiable, encrypted dataset will be exported from REDCap and all injury data collected during the study period will be analysed. Descriptive and analytical analysis will be performed using SPSS [35]. Analysis will be conducted to describe patterns and outcomes of injury using basic frequencies, 95% confidence intervals, means and standard deviations. Where feasible and appropriate, associations between socio-demographics, patterns of injury and severity will be explored using multivariable regression analysis, reporting through odds ratios (OR), *P* value (0.05 significant level) and confidence intervals (CI 95%).

#### Qualitative data

Audio recordings of interviews will be downloaded onto a password-protected computer in the MIRA office as soon as possible and the digital recording deleted. The electronic recording will be transcribed verbatim and translated from Nepali to English. The transcript and translated copy will be cross-checked to ensure the accuracy of the translations. Personal identification information for all participants will be removed whilst transcribing the recording and replaced with a unique identification code. Qualitative data will be analysed thematically using codes generated iteratively through the reading and re-reading of transcripts [36]. A random sample of transcripts will be coded by two researchers, if there is any disagreement, codes will be discussed with a third researcher to agree a coding framework. The coding framework will then be applied across all transcripts and themes relating to the facilitators and barriers to effective injury surveillance will be identified. The analysis will be supported by the NVivo Qualitative Data Analysis Software, Version 11 [37].

### Ethical considerations

Patients will be told about the study once their urgent care needs have been met and asked if they are willing to have anonymised information about their injury recorded. In this study consent will therefore be sought verbally from patients prior to data collection and their response recorded on REDCap. For this study, this method of consent has been approved by the Ethical Review Board of Nepal Health Research Council and from the Faculty Research Ethics Committee of University of the West of England, Bristol. The study protocol and delivery of this study will adhere to guidance provided by the WHO’s ‘Guidelines on Ethical Issues in Public Health Surveillance’ and in particular to the four overriding principles underpinning the guidelines: (i) Common good; (ii) Equity; (iii) Respect for persons; (iv) Good governance [38]. The data collection forms and database included in this study will ensure that all patient information and injury details will be uncoupled from any personal information or identifiers prior to any data leaving hospital sites. The data collected in this study will not alter or interfere with patient care in any way.

Ethical approval was obtained from the Ethical Review Board of Nepal Health Research Council and from the Faculty Research Ethics Committee of University of the West of England, Bristol. Written permission to access clinical information from the hospitals involved in the study was obtained from each hospital’s clinical management board and from local government representatives in Makwanpur District, Nepal.

## Discussion

To our knowledge, this is the first attempt to establish and comprehensively study the design and implementation of a multi-site hospital-based injury surveillance programme in Nepal and could enable further work on establishing an extended injury surveillance system in Nepal. We anticipate collecting injury data from around 10,000 patients in this study, making it the largest prospective hospital-based injury data surveillance collection ever undertaken in Nepal. The effective use of injury surveillance data in Nepal could support the reduction in morbidity and mortality from adult and childhood injury through improved understanding of trauma patterns and epidemiology as well as injury prevention intervention development, resource allocation and policy development.

A recent focus on advancing research on emergency care systems in low and middle income countries has again highlighted the importance of novel methodologies and study protocols for examining key emergency care questions, especially in relation to data sources that can be used to influence policy and modify the delivery of clinical care [39, 40]. In addition, emergency care surveillance has been specifically highlighted as a needed and neglected area of focus in most low and middle income settings, with injury surveillance requiring special attention and a need for addressing exactly the kind of opportunities and challenges which have been included in the design of this study protocol [41]. For example, moving from cross sectional to surveillance data capture, engaging local stakeholders and partners, using electronic data capture, and developing a culture of data use. Trauma registry data captured from embedded clinical data instruments for clinical staff is an alternative to more traditional injury surveillance carried out by specific data collectors (as in this study). However significant challenges exist with relying on clinical staff to collect injury data including clinical staff capacity, the quality of data, lack of resources, insufficient prehospital care, and difficulty with administrative duties and hospital organisation [27]. Such challenges can undermine the value of the data collected as evidenced through one recent, well organised study in Ethiopia which found a capture rate of just 21% for injury data on their clinical data tool [31].

A number of challenges exist in relation to collecting injury data in the clinical environment and this study protocol outlines a number of key components for generating what we hope will be a generalisable approach for establishing injury surveillance of this kind: Training and engagement with local health and government actors including the emergency departments with whom we are working; use of real time, electronic data capture and internet based data uploads; designated data collection staff (with a health background) working alongside clinical staff; prospective data collection over 12 months; information gathering on a focused group of injury data fields; and, use of interviews and qualitative data to inform further development of the surveillance system. The recruitment of data collection staff with experience of working in health settings is of particular importance as this enables a degree of comfort with working in the emergency department setting, familiarity with the more clinical components of data collection relating to injuries and an element of ‘psychological safety’ when exposed to patients with more severe of life threatening injuries. Tablet computers were funded for this study as there was no system available to capture patient data electronically in the department. Ownership of mobile phones (including smart phones) is increasing rapidly, and mobile phone coverage in Nepal is now excellent. Increasingly internet access is also good, suggesting that such methods might be increasingly feasible in Nepal. In addition, we have reflected on the importance of a thorough and context specific understanding of the urgent care pathway, patient flow and clinical processes in the setting in which data collection is to take place. For example prior to the data collection described in this protocol we did not appreciate that patients with dog bites were routed to a separate outpatient area of Hetauda hospital rather than the emergency department – something to which we were fortunately able to respond with respect to capturing data on these patients.

The primary limitation of hospital injury surveillance systems is that of selection bias because it will only identify people with injuries presenting to hospitals and will therefore miss patients seeking care from community-based health services, local healers or providing self-care. In addition, the data will only capture patients self-reporting with injuries and will not be linked to any prehospital data system. Our study will therefore underestimate the true burden of injuries in the communities being studied. It will potentially also miss injury related morbidity and mortality where the injury mechanism occurred more than 7 days before the hospital presentation as well as those in the community that do not come to the hospital. Regarding injury deaths specifically, our anecdotal experience is that most patients who have died from trauma in this region are brought to the hospital as part of police investigations and for post-mortem examination. These facts should be considered when interpreting the data and generalising the findings beyond the study setting. A strength of the study design is that this study will collect all injury cases reported over a period of 12 months and will therefore not be subject to seasonal variations in injury incidence (for example drowning associated with monsoon flooding).

This study seeks to generate new and novel knowledge regarding the burden of injuries affecting a community living in a district in southern Nepal. In addition, it will test a system of hospital-based injury surveillance in both a government and private hospital in Nepal and explore the opportunities to establish a sustainable system of injury surveillance at the study sites and the potential to expand the model to additional hospitals creating a multi-site injury surveillance system in Nepal.

## Data Availability

Data sharing is not applicable to this article as no datasets were generated or analysed during the development of the study protocol.

## Abbreviations

DALY: Disability Adjusted Life Year
ED: Emergency Department
HASS: Home and Leisure Accident and Surveillance System
HMIS: Hospital Management Information System
KMC: Kathmandu Medical College
LMIC: Low and middle income countries
MIRA: Mother and Infant Research activities
NIRC: Nepal Injury Research Centre
OR: Odds Ratio
REDCap: Research Electronic Data Capture
WHO: World Health Organization
